# Estimated Cost of Transcarotid Arterial Revascularization Compared With Carotid Endarterectomy and Transfemoral Carotid Stenting

**DOI:** 10.7759/cureus.23539

**Published:** 2022-03-27

**Authors:** John J Kanitra, Isabella A Graham, Richard D Hayward, Darla K Granger, Richard A Berg, Jimmy C Haouilou

**Affiliations:** 1 Department of Surgery, Ascension St. John Hospital, Detroit, USA

**Keywords:** carotid endarterectomy, carotid stent, hospital costs, carotid revascularization costs, transcarotid artery revascularization (tcar)

## Abstract

Objectives

Transcarotid arterial revascularization (TCAR) is associated with a lower risk of stroke or death than transfemoral carotid artery stenting (TF-CAS). TCAR infers a lower risk of cranial nerve injury and a similar risk of myocardial infarction (MI) than carotid endarterectomy (CEA). There have been no comparative studies on the cost of TCAR, TF-CAS, and CEA, which may have important implications for institutional support for the new modality to address carotid artery stenosis. Our aim was to compare the estimated cost profiles of TCAR, TF-CAS, and CEA.

Methods

A review was performed on Medicare patients who underwent TCAR, TF-CAS, or CEA between January 1, 2020, and December 31, 2020. Demographics, comorbidities, operative details, and postoperative complications were reviewed. Acute stroke presentations and elective procedures were included. Cost data were obtained from the hospital’s finance department. Quantitative variables were compared using analysis of variance, and categorical variables were compared using the chi-square analysis.

Results

In total, 21 TCAR, 97 TF-CAS, and 26 CEA patients were initially identified. After removing the non-Medicare patients, 17 TCAR, 57 TF-CAS, and 13 CEA patients were included in the analysis. In-hospital stroke, MI, and mortality included three deaths in TF-CAS patients. At 30 days, the stroke rates for TCAR, TF-CAS, and CEA groups were 0%, 1.8%, and 0%, respectively.

The payments for TCAR, TF-CAS, and CEA were $15,400 ± 2,100, $23,400 ± 11,800 and $14,300 ± 5,700 (p=0.001), respectively. The estimated costs for TCAR, TF-CAS, and CEA were $10,500 ± 3,300, $13,800 ± 14,300, and $12,400 ± 6,000 (p=0.575), respectively. The profit margins for TCAR, TF-CAS, and CEA were $5,100 ± 3,100, $9,600 ± 12,100, and $1,900 ± 6,400 (p=0.032), respectively.

There was no significant difference in American Society of Anesthesiologists (ASA) scores (p=0.635) or age (p=0.485) among the three groups. The length of hospital stay was not significantly different (p=0.107).

The TF-CAS maintained the highest profit margin (p<0.001) when matched for the same diagnosis-related code (without complications or comorbidities).

Urgency classification within the TF-CAS group included 45 elective, four urgent, and eight emergent cases. The profit margin was significantly higher for the elective group than for the emergent group (p=0.002) but not different for elective versus urgent (p=0.503) or urgent versus emergent (p=0.102). All patients who underwent TCAR and CEA were elective.

Conclusion

The hospital reimbursement and profit margins are higher for TF-CAS than for TCAR. With the increasing data now demonstrating similar outcomes with TF-CAS and CEA, further research is required to examine the long-term cost-effectiveness of TCAR and how this will compare to TF-CAS.

## Introduction

Carotid stents, traditionally placed via the transfemoral route, are generally reserved for patients at a high risk for carotid endarterectomy (CEA). Transfemoral carotid artery stenting (TF-CAS) can be complicated by a distal plaque embolism, incurring a 30-day stroke rate of 4% [1]. Transcarotid arterial revascularization (TCAR) was developed to mitigate this complication. This is achieved by reversing blood flow through the carotid artery via an ex vivo carotid artery to a femoral vein shunt while a carotid stent is placed. In theory, any plaque disruption would embolize to the external appliance as opposed to intracranially. Additionally, it obviates the risk of passing a wire through an atherosclerotic aorta, as in TF-CAS. As intended, current retrospective data demonstrate a lower stroke rate with TCAR than with TF-CAS [2]. A similar stroke rate of 1-2% is seen between CEA and TCAR despite higher risk comorbidities in TCAR patients [3,4]. The stroke rate between TCAR and CEA remained similar at one year. That is, 2-3% [4].

The cost of TF-CAS is 50-70% higher compared with CEA [5]. There is limited literature on the cost for TCAR. Cui et al. suggested that the five-year cost for TCAR is approximately $10,000 more than that for CEA, but TCAR is associated with more quality-adjusted life years (QALYs) [6]. A recent cost-effectiveness study found CEA to be more cost-effective than TCAR and TF-CAS [7]. The cost of TCAR may have important implications for gaining institutional support for this new modality. We aimed to examine the cost profile of TCAR compared with CEA and TF-CAS at a single institution.

## Materials and methods

This study met the criteria for exempt research by the Ascension St. John Hospital Institutional Review Board, and the requirement for written consent was waived.

A review was performed on TCAR, TF-CAS, and CEA patients from January 1, 2020, to December 31, 2020. Patients were included if they had Medicare or Medicare Advantage insurance plans. Patients with TF-CAS and TCAR were identified from the Society for Vascular Surgery Vascular Quality Initiative (VQI) regional database. Additional information can be found at www.vascularqualityinitiative.org and ClinicalTrials.gov (NCT02850588). Our institution does not participate in the CEA VQI. CEA patients were identified using the Blue Cross Blue Shield of Michigan Cardiovascular Consortium Regional Database (BMC2 PCI), which includes all Medicare patients regardless of their use of the Blue Cross insurance agency. Additional information can be found at www.bmc2.org. Data reviewed for TCAR, TF-CAS, and CEA included patient age, American Society of Anesthesiologists (ASA) score, and length of stay. The demographics, comorbidities, operative details, and postoperative complications were also reviewed. A retrospective chart review of our institutional electronic medical records was performed to confirm myocardial infarction (MI), stroke, or death events. Additionally, the chart review was used to abstract the demographic data, which was not available in the BMC2 PCI.

Urgency classifications were obtained from the respective databases. BMC2 defines emergent as operation within 12 hours of symptoms, urgent as operation within 72 hours of symptoms, and elective as all others. The VQI defines emergent as operation within six hours of admission, urgent as surgery within 24 hours, or if the patient cannot be discharged and elective as all others.

Patients were excluded from the study if they had non-Medicare insurance to avoid confounders with different payment criteria and reimbursement amounts. Patient charges and insurance payments were obtained from an institutional business objects database, which obtains data from the institution’s accounting department. The estimated hospital costs were determined by assessing the patient charges based on revenue codes. Each revenue code corresponds to a Medicare category (i.e., operating room, recovery room, room and board, etc.), each of which has a unique cost-to-charge ratio. The total estimated cost for hospital stay was calculated by taking the sum of the charges for each patient, separating it based on the revenue code, and applying the corresponding Medicare cost-to-charge ratio to each revenue code. A weighted mean of the resulting value was then obtained based on the number of patients who had charges in that code. Device-specific costs were assessed by comparing the institutional price of the TCAR system (SilkRoad Medical Inc., Sunnyvale, CA) and a carotid stent (Abbott Cardiovascular Inc., Chicago, IL; embolic protection, SpiderFX™ embolic protection device, Medtronic, Minneapolis, MN). Institutional policy prohibits us from listing the price of each device.

Statistical analysis 

Continuous variables (including hospital cost and charge data) are described as mean with standard deviation. Categorical variables are described as frequencies. Univariate analyses of factors associated with stroke, MI, and death were performed using analysis of variance (ANOVA) or chi-square analyses to compare patients by treatment category. Analyses were conducted using SAS 9.4 (SAS Institute Inc., Cary, NC).

## Results

In total, 21 TCAR, 97 TF-CAS, and 26 CEA patients were initially identified. After removing the non-Medicare patients, 17 TCAR, 57 TF-CAS, and 13 CEA patients were included in the analysis. There were no outstanding balances from any Medicare supplemental plan. In-hospital stroke, MI, or mortality included three deaths in patients with TF-CAS. The patient demographics and comorbidities are summarized in Table 1.

**Table 1:** Patient demographics and comorbidities ^*^13 patients were available for one-year follow-up. ^†^43 patients were available for one-year follow-up. Three additional patients experienced a mortality of >30 days postoperatively. One patient underwent balloon angioplasty of the carotid stent 307 days after insertion. ^‡^One patient had 50-69% stenosis, and 10 had 70-99% stenosis. Two patients had these data missing on chart review. ^§^11 patients were available for one-year follow-up. TCAR, transcarotid arterial revascularization; TF-CAS, transfemoral carotid artery stenting; CEA, carotid endarterectomy; MI, myocardial infarction

	TCAR	TF-CAS	CEA
Total, n	17	57	13
Female, n (%)	3 (17.7)	28 (49.1)	7 (53.8)
Age, years, mean ± SD	73.5 ± 5.7	75.6 ± 7.5	77.0 ± 4.7
Past medical history			
Diabetes, n (%)	4 (23.5)	26 (45.6)	4 (30.8)
Current smoker, n (%)	4 (23.5)	9 (15.8)	2 (15.4)
Past smoker, n (%)	8 (47.1)	29 (50.9)	7 (53.8)
Prior ipsilateral carotid intervention, n (%)	1 (5.9)	2 (3.5)	3 (21.4)
Prior contralateral carotid intervention, n (%)	3 (17.6)	4 (7.0)	4 (30.8)
Operative details			
Symptomatic disease, n (%)	2 (11.8)	30 (52.6)	1 (7.7)
Laterality: right, n (%)	9 (52.9)	35 (61.4)	4 (30.8)
Percent stenosis, mean ± SD	86.2 ± 4.5	84.7 ± 10.7	‡
Time of flow reversal, mean ± SD	16.8 ± 5.8		
Postoperative			
30-day mortality, n (%)	0	3 (5.3)	0
One-year mortality, n (%)	1 (5.9)^*^	6 (14)^ †^	0^§^
30-day stroke, n (%)	0	1 (1.8)	0
One-year stroke, n (%)	0^*^	0^†^	1 (9.1)^§^
30-day MI, n (%)	0	0	0
One-year MI, n (%)	0^*^	1 (2.3)^†^	0^§^

More than 80% of the patients had hyperlipidemia and hypertension. The 30-day and one-year mortality, stroke, and MI rates are shown in Table 1. Two TCAR patients had two stents placed, and the remaining TCAR and TF-CAS patients had one stent placed.

The cost profiles of TCAR, TF-CAS, and CEA are listed in Table 2.

**Table 2:** Cost profiles broken down by diagnosis-related groups Data are represented as mean ± standard deviation. Values in parentheses represent ranges. Monetary values are given in 1,000 U.S. dollars. The p-values are for ANOVA for mean differences between groups in the corresponding variables, with the exception of ICU stay, for which p-values are for chi-square tests of independence. *All were elective procedures TCAR, transcarotid arterial revascularization; TF-CAS, transfemoral carotid artery stenting; CEA, carotid endarterectomy; ASA, American Society of Anesthesiologists; ICU, intensive care unit

	TCAR (n=17)	TF-CAS (n=57)	CEA (n=13)	p-Value
Profit margin ($)	5.1 ± 3.1 (-3.5 to 10.9)	9.2 ± 12 (-19.9 to 39.9)	1.9 ± 6.4 (-5.0 to 18.3)	0.046
Cost ($)	10.5 ± 3.3 (7.7–20.7)	13.8 ± 14.3 (3.2–63.0)	12.4 ± 6.0 (6.5–28.5)	0.575
Charges ($)	55.3 ± 15.5 (40.3–107.1)	66.8 ± 52.1 (22.7–175.9)	61.4 ± 22.6 (36.8–116.2)	0.624
Payment ($)	15.4 ± 2.1 (13.1–19.1)	22.9 ± 11.8 (11.7–56.7)	14.3 ± 5.7 (8.7–25.8)	0.002
Length of stay (days)	1.4 ± 1.1 (1.0–1.5)	4.6 ± 6.9 (0.0–33.0)	2.5 ± 2.2 (1.0–4.0)	0.107
ASA scores	3.2 ± 0.6 (2.0–4.0)	3.2 ± 0.6 (2.0–4.0)	3.8 ± 0.5 (2.0–4.0)	0.635
ICU stay (n)	5	11	4	0.528
ICU days	1.8 ± 0.8	4.2 ± 6	2.5 ± 1	0.582
Without complications and comorbidities^*^				
n	11	21	3	
Profit margin ($)	4.9 ± 1.1	9.5 ± 2.7	-0.3 ± 1.2	0.007
Cost ($)	9.1 ± 0.7	5.0 ± 1.7	9.3 ± 1.5	<0.001
Charges ($)	50.8 ± 4.0	32.5 ± 10.7	51.7 ± 5.3	<0.001
Payment ($)	14.0 ± 0.7	14.5 ± 2.8	9.0 ± 0.3	0.001
Length of stay (days)	1.0 ± 0.0	1.1 ± 0.5	1.3 ± 0.6	0.524
ASA scores	3.3 ± 0.6	2.8 ± 0.5	3.7 ± 0.6	0.018
ICU stay (n)	1	2		0.999
ICU days	1	1.5 ± 0.7		0.667
With complications and comorbidities				
n	6	21	8	
Profit margin ($)	5.3 ± 3.3	8.5 ± 9.5	14.1 ± 5.0	0.118
Cost ($)	12.6 ± 4.9	12.8 ± 6.9	12.1 ± 4.0	0.967
Charges ($)	63.5 ± 24.8	70.0 ± 42.0	60.8 ± 18.5	0.801
Payment ($)	17.9 ± 1.0	21.3 ± 8	13.5 ± 3.1	0.023
Length of stay (days)	2.2 ± 1.6	4.5 ± 6.8	2.3 ± 1.0	0.477
ASA scores	3.2 ± 0.4	3.8 ± 0.5	3.1 ± 0.4	0.327
ICU stay (n)	3	3	3	0.273
ICU days	2 ± 1	3 ± 2	2 ± 0	0.960
With major complications and comorbidities				
n	0	15	2	
Profit margin ($)		9.6 ± 20.8	7.1 ± 1.6	0.873
Cost ($)		27.5 ± 20.7	18.0 ± 14.9	0.546
Charges ($)		110.5 ± 66.0	78.7 ± 53.1	0.526
Payment ($)		37.1 ± 11.3	25.1 ± 1.0	0.164
Length of stay (days)		9.5 ± 9.0	5.0 ± 5.7	0.512
ASA scores		3.5 ± 0.6	4.0 ± 0.0	0.332
ICU stay (n)		4	1	0.643
ICU days		8 ± 9.3	4	0.761

There was a significant difference between the profit margins of the three groups (p=0.032), with TF-CAS having the highest margin. There was no significant difference in ASA scores (p=0.635) or age (p=0.485) among the three groups. The length of stay was not significantly different (p=0.107). A supplemental analysis eliminating the three mortality cases for TF-CAS did not change the significance of any results, although the average total hospital cost for TF-CAS decreased (Table 3). Another analysis was performed comparing elective only patients in all three groups, which again continued to demonstrate the highest profit-margin with TF-CAS (Table 3).

**Table 3:** Cost profiles of non-mortality cases and elective only cases Data are represented as mean ± standard deviation. Values in parentheses represent ranges. Monetary values are given in 1,000 U.S. dollars. The p-values are for ANOVA for mean differences between groups in the corresponding variables, with the exception of ICU stay, for which p-values are for chi-square tests of independence. TCAR, transcarotid arterial revascularization; TF-CAS, transfemoral carotid artery stenting; CEA, carotid endarterectomy; ASA, American Society of Anesthesiologists; ICU, intensive care unit

	TCAR	TF-CAS	CEA	p-Value
Excluding mortality cases				
n	17	54	13	
Profit margin ($)	5.1 ± 3.1 (-3.5 to 10.9)	10.1 ± 11.5 (-19.9 to 39.9)	1.9 ± 6.4 (-5.0 to 18.3)	0.013
Cost ($)	10.5 ± 3.3 (7.7–20.7)	11.9 ± 11.4 (3.2–74.2)	12.4 ± 6.0 (6.5–28.5)	0.803
Charges ($)	55.3 ± 15.5 (40.3–107.1)	59.2 ± 38.1 (22.7–291.9)	61.4 ± 22.6 (36.8–116.2)	0.867
Payment ($)	15.4 ± 2.1 (13.1–19.1)	22.0 ± 11 (12.2–56.7)	14.3 ± 5.7 (8.7–25.8)	0.005
Length of stay (days)	1.4 ± 1.1 (1.0–1.5)	4.1 ± 6.7 (0.0–33.0)	2.5 ± 2.2 (1.0–4.0)	0.180
ASA scores	3.2 ± 0.6 (2.0–4.0)	3.2 ± 0.6 (2.0–4.0)	3.8 ± 0.5 (2.0–4.0)	0.479
ICU stay (n)	5	9	4	0.360
ICU days	1.8 ± 0.8	2.4 ± 1.4	2.5 ± 1	0.593
Elective cases only				
n	17	45	13	
Margin ($)	5.1 ± 3.1 (-3.5 to 10.9)	10.7 ± 9.7 (-6.3 to 39.9)	1.9 ± 6.4 (-5.0 to 18.3)	0.001
Cost ($)	10.5 ± 3.3 (7.7–20.7)	9.7 ± 7.8 (3.2–35.2)	12.4 ± 6.0 (6.5–28.5)	0.455
Average charges ($)	55.3 ± 15.5 (40.3–107.1)	54.3 ± 37.5 (22.7–202.4)	61.4 ± 22.6 (36.8–116.2)	0.773
Average payment ($)	15.4 ± 2.1 (13.1–19.1)	20.4 ± 10.1 (11.7–53.6)	14.3 ± 5.7 (8.7–25.8)	0.022
Length of stay (days)	1.4 ± 1.1 (1.0–1.5)	3.0 ± 5.2	2.5 ± 2.2 (1.0–4.0)	0.398
ASA score	3.2 ± 0.6 (2.0–4.0)	3.1 ± 0.6	3.8 ± 0.5 (2.0–4.0)	0.238
ICU stay (n)	5	8	4	0.463
ICU days	1.8 ± 0.8	2.3 ± 1.4	2.5 ± 1	0.662

Five TCAR, 11 TF-CAS, and four CEA patients had an intensive care unit (ICU) stay during the index hospital admission. Of the five TCAR patients requiring an ICU stay, two had bradycardia, four had hypotension, and two had a cardiology consultation to assist in the management of the aforementioned symptoms. Of the 11 TF-CAS patients, seven had bradycardia, 10 had hypotension, six required central line access for vasopressors, and eight had cardiology consults. Of the four CEA patients, one patient returned to the operating room for hematoma evacuation, one developed hypotension and bradycardia, one had bradycardia alone, and the fourth patient was monitored for concern of postoperative stroke. All four CEA patients had a cardiology consultation.

Table 2 also demonstrates the breakdown based on the diagnosis-related group, which is a grouping based on patient ICD-10 diagnosis and procedure codes. The patients in the diagnosis-related group “without complications and comorbidities” were all elective cases, and therefore did not include any acute stroke patients. TF-CAS patients without complications or comorbidities had a significantly higher profit margin than those with both TCAR and CEA. The difference between TF-CAS and TCAR is a result of the significantly lower cost of TF-CAS compared to TCAR. No differences were observed in the patients with complications and comorbidities.

The urgency classification within the TF-CAS group included 45 elective, four urgent, and eight emergent cases (Table 4).

**Table 4:** TF-CAS cost profile based on urgency Data are represented as mean ± standard deviation. Values in parentheses represent ranges. Monetary values are given in 1,000 U.S. dollars. ASA, American Society of Anesthesiologists; TF-CAS, transfemoral carotid artery stenting

	Elective	Urgent	Emergent
n	45	4	8
Margin ($)	10.7 ± 9.7 (-6.3 to 39.9)	15.0 ± 17.6 (1.6–39.8)	-2.6 ± 15.1 (-19.9 to 18.6)
Cost ($)	9.7 ± 7.8 (3.2–35.2)	14.3 ± 1.2 (13.4–16.1)	36.5 ± 23.8 (12.4–74.2)
Average charges ($)	54.3 ± 37.5 (22.7–202.4)	74.1 ± 27.0 (46.0–104.3)	133.7 ± 80.1 (47.8–291.9)
Average payment ($)	20.4 ± 10.1 (11.7–53.6)	29.3 ± 17.0 (17.3–53.3)	33.9 ± 11.9 (17.9–56.7)
Length of stay (days)	3.0 ± 5.2	3.5 ± 1.3	13.6 ± 10.1
ASA score	3.1 ± 0.6	3.8 ± 0.5	3.6 ± 0.5

The profit margin was significantly higher for the elective group than for the emergent group (p=0.002) but not different for elective versus urgent (p=0.503) or urgent versus emergent (p=0.102). All patients who underwent TCAR and CEA were elective.

The cost breakdown of the major departments contributing to the total hospital costs of the patients is shown in Table 5.

**Table 5:** Cost breakdown per patient Data are represented as mean ± standard deviation. Values in parentheses represent ranges. Monetary values are given in 1,000 U.S. dollars. *The large differences in this cost are largely explained by computed tomography (CT) scans. In total, 20 TF-CAS patients and one CEA patient underwent a CT. TCAR, transcarotid arterial revascularization; TF-CAS, transfemoral carotid artery stenting; CEA, carotid endarterectomy; ICU, intensive care unit

	TCAR	TF-CAS	CEA
Floor ($)	1,592 ± 783	5,555 ± 5,564	2,687 ± 1,586
ICU ($)	3,525 ± 1,110 (n=4)	7,718 ± 11,981 (n=12)	4,246 ± 2,050 (n=4)
Operating room/endovascular suite ($)	5,717 ± 681	3,860 ± 984	5,554 ± 1,381
Postoperative recovery room ($)	1,338 ± 418	623 ± 274	1,250 ± 457
Anesthesia ($)	312 ± 97	284 ± 165	479 ± 180
Radiology^*^ ($)	3 ± 5	4,381 ± 523	67 ± 53
Pharmacy ($)	486 ± 21	607 ± 58	565 ± 21
Laboratory ($)	123 ± 13	372 ± 74	239 ± 26
Emergency department ($)	n/a	577 ± 238	n/a
Cost breakdown per patient for elective procedures in the diagnosis-related group “without complications or comorbidities”			
n	11	21	3
Floor ($)	1,314 ± 260	1,690 ± 1,105	1,667 ± 308
ICU ($)	2,564 (n=1)	2,403 ± 277 (n=2)	0
Operating room/endovascular suite ($)	5,432 ± 577	2,666 ± 312	5,358 ± 1,323
Postoperative recovery room ($)	1,428 ± 420	424 ± 344	1,172 ± 388
Anesthesia ($)	285 ± 74	189 ± 42	443 ± 163
Radiology^*^ ($)	1 ± 3	2,405 ± 380 (n=5)	0
Pharmacy ($)	446 ± 23	169 ± 8	407 ± 16
Laboratory ($)	107 ± 11	95 ± 13	221 ± 36
Emergency department ($)	n/a	n/a	n/a

The numbers provided in Table 5 represent the estimated costs per patient. Notably, not all patients had charges in each revenue category; therefore, the total hospital cost cannot be calculated simply by the sum of these values. The weighted mean was calculated (see the Methods section). To control for urgency of the procedure and diagnosis-related groups, Table 5 also provides the cost breakdown of the elective procedures in the diagnosis-related group “without complications and comorbidities,” as found in Table 2. The TCAR-specific devices (stent and flow reversal device) cost approximately $2,700 more than the TF-CAS-specific devices (stent and embolic protection devices).

## Discussion

Multiple trials and VQI studies compared the outcomes of TCAR, TF-CAS, and CEA but only one compared the cost-effectiveness between them using a Markov model [7]. Another looked at cost for TCAR compared to CEA [6]. We aimed to look at cost during the index hospital admission, as well as insurance payments. At our institution, we demonstrated no significant difference in the costs of the three procedures; however, the profit margin for index hospital admission was the highest for TF-CAS.

The stroke pathway at our institution includes deployment of a neuroendovascular team that performs a cerebral angiogram if indicated. A carotid stent is placed during the cerebral angiogram if carotid stenosis is identified. Vascular surgery is not routinely involved in the stroke pathway, which led to a high proportion of TF-CAS observed in the current study. It is likely that other institutions are seeing similar trends since there has been a significant increase in TF-CAS volume in the post-CREST (Carotid Revascularization Endarterectomy Versus Stenting Trial) era [8].

CREST compared CEA and TF-CAS [9]. There was no difference in CEA and TF-CAS in regard to the primary endpoint: the composite of any stroke, MI, or death during the periprocedural period or ipsilateral stroke within four years. However, periprocedural stroke was lower in CEA than in TF-CAS (2.3% vs. 4.1%, p=0.01), and MI was higher in CEA (2.3% vs. 1.1%, p=0.03). Cole et al. examined the Nationwide Readmissions Database in the subsequent six years following the CREST trial, matching patients for characteristics and illness severity, and found that patients undergoing CEA actually had a higher perioperative stroke than TF-CAS (2.6% vs. 1.9%, p<0.001), in contrast to CREST [8]. This may be a result of increased surgeon experience and progress in endovascular methods. Of note, the proportion of symptomatic patients was higher in the CREST (47%) than in the study by Cole et al. (30%).

Although the initial evidence demonstrates a higher stroke rate in TF-CAS compared to TCAR and CEA [2,9], the recent literature is increasingly supportive of TF-CAS. Ten-year follow-up of CREST patients did not find a significant difference in ipsilateral stroke between TF-CAS and CEA [10]. The recent Asymptomatic Carotid Stenosis Trial (ACST-2) added more evidence that the outcomes of TF-CAS and CEA are similar with a five-year follow-up [11].

Medicare will reimburse for a TF-CAS in symptomatic patients if they are at high surgical risk and have greater than 70% carotid stenosis or 50-70% stenosis if the patient is enrolled in an approved clinical trial. Asymptomatic patients must have greater than 80% carotid stenosis and be enrolled in an approved clinical trial [12]. Medicare coverage for TCAR follows the same guidelines as TF-CAS. Currently, the approved trials are VQI-TCAR (NCT02850588), ROADSTER 2 (NCT02536378), and CREST-2 (NCT02089217).

The existing literature suggests a higher cost for TF-CAS at index hospital admission but similar long-term costs compared to CEA. We demonstrated an 11% increase in the average cost for index hospital admission for TF-CAS than CEA compared to the published 40% [13]. In Cole et al.’s cohort, CEA had a lower inpatient cost ($14,433 vs. $19,172, p<0.001) than TF-CAS when controlling for illness severity without a difference in length of stay [8]. A 2016 British randomized control trial with 1,700 patients between TF-CAS and CEA with a 10-year follow-up found no difference in costs between the two modalities at index admission and total cost including QALYs [14]. A 2017 meta-analysis looked at the inpatient cost between CEA and TF-CAS and found a higher procedural cost for TF-CAS but similar inpatient and long-term costs [15]. A 2017 retrospective study of approximately 30,000 patients in the United States found higher inpatient costs for TF-CAS compared to CEA, despite matching patients, supporting the results of Cole et al. [13]. However, since postprocedure costs are lower with TF-CAS, projected 10-year outcomes suggest a negligible difference in cost between the two modalities [16].

Cui et al. performed a cost-effectiveness analysis comparing TCAR and CEA using a simulation model of 10,000 symptomatic patients over a five-year period. Cost data were derived from peer-reviewed sources in the literature. The five-year cost was approximately $10,000 more for TCAR than CEA, but TCAR afforded greater QALYs and became cost-effective at six years [6]. More recently, Sridharan et al. performed a cost-effectiveness analysis using a Markov model to compare TCAR, TF-CAS, and CEA. They found a TCAR cost of $160,642 per QALY compared to CEA, which is over the willingness-to-pay threshold of $100,000 per QALY. TCAR could become cost-effective compared to CEA if the risk of stroke following TCAR could be reduced to <0.9% or if the procedure cost could be decreased by $2,000. TF-CAS was the least cost-effective of all three modalities due to the highest stroke risk [7]. The current study demonstrated an approximately 31% increase in the average cost for TF-CAS compared with TCAR at index hospital admission. Perhaps, TCAR is a superior procedure in terms of cost, although a true cost-benefit study needs to be performed.

We found room and board (floor and/or ICU) and the operating suite to be the largest drivers of cost for the index hospital admission (Table 5), which is not necessarily directly a result of the procedure itself. This is largely the reason CEA demonstrated a non-significantly higher cost than TCAR, even though there was no device (flow reversal) in CEA. Overall, TF-CAS had the largest proportion of patients with an ICU stay, as expected, given the acute stroke cases, contributing to a higher cost than TCAR (Table 2). TF-CAS had the highest radiology cost due to computed tomography scans obtained during a stroke workup. When controlling for the urgency of cases and complications or comorbidities, the operating room cause was considerably less for TF-CAS than for TCAR and CEA, reflective of lower costs of an endovascular suite compared to an operating room.

The limitations of the current study include its single center, retrospective design, and a small cohort. The length of stay, proportion requiring an ICU stay, and number of symptomatic patients were higher with TF-CAS, all of which could impact the higher reimbursement. However, we examined diagnosis-related codes to try to mitigate this confounder and found a higher profit margin for TF-CAS when matched for the same diagnosis-related group (without complications and comorbidities). All patients across the three procedures in this diagnosis-related group were elective. Therefore, this comparison was done with the most similar patients. We used the Medicare cost-to-charge ratio to estimate the cost, which is dependent on how our institution charges a patient and is not necessarily an accurate representation of the true cost. A more accurate method is to use cost accounting, which is unfortunately only available for institutional use. Although our data may not reflect the true cost, the current study compares the cost data between the three procedures, as opposed to drawing conclusions from a single value. Therefore, confounders should be consistent across the data for all procedures.

## Conclusions

The current study compared the cost of the three modalities used to manage carotid stenosis at index hospital admission. Our data suggest that the hospital reimbursement and profit margins are higher for TF-CAS than for TCAR. When controlling for urgency and complications/comorbidities, TF-CAS had the highest profit margin predominately from the lower cost of the endovascular suite compared to an operating room. With the increasing data now demonstrating similar outcomes with TF-CAS and CEA, further research is required to examine the long-term cost-effectiveness of TCAR and how this will compare to TF-CAS.
